# A Novel Method to Adjust Efficacy Estimates for Uptake of Other Active Treatments in Long-Term Clinical Trials

**DOI:** 10.1371/journal.pone.0008580

**Published:** 2010-01-08

**Authors:** John Simes, Merryn Voysey, Rachel O'Connell, Paul Glasziou, James D. Best, Russell Scott, Christopher Pardy, Karen Byth, David R. Sullivan, Christian Ehnholm, Anthony Keech, for the FIELD Study Investigators

**Affiliations:** 1 National Health and Medical Research Council Clinical Trials Centre, University of Sydney, Sydney, Australia; 2 Centre for Evidence-Based Medicine, University of Oxford, Oxford, United Kingdom; 3 Faculty of Medicine, University of Melbourne, Melbourne, Australia; 4 Lipid and Diabetes Research Group, Christchurch Hospital, Christchurch, New Zealand; 5 Department of Clinical Biochemistry, Royal Prince Alfred Hospital, Sydney, Australia; 6 Biomedicum Helsinki, National Public Health Institutes, Helsinki, Finland; University of South Florida, United States of America

## Abstract

**Background:**

When rates of uptake of other drugs differ between treatment arms in long-term trials, the true benefit or harm of the treatment may be underestimated. Methods to allow for such contamination have often been limited by failing to preserve the randomization comparisons. In the Fenofibrate Intervention and Event Lowering in Diabetes (FIELD) study, patients were randomized to fenofibrate or placebo, but during the trial many started additional drugs, particularly statins, more so in the placebo group. The effects of fenofibrate estimated by intention-to-treat were likely to have been attenuated. We aimed to quantify this effect and to develop a method for use in other long-term trials.

**Methodology/Principal Findings:**

We applied efficacies of statins and other cardiovascular drugs from meta-analyses of randomized trials to adjust the effect of fenofibrate in a penalized Cox model. We assumed that future cardiovascular disease events were reduced by an average of 24% by statins, and 20% by a first other major cardiovascular drug. We applied these estimates to each patient who took these drugs for the period they were on them. We also adjusted the analysis by the rate of discontinuing fenofibrate. Among 4,900 placebo patients, average statin use was 16% over five years. Among 4,895 assigned fenofibrate, statin use was 8% and nonuse of fenofibrate was 10%. In placebo patients, use of cardiovascular drugs was 1% to 3% higher. Before adjustment, fenofibrate was associated with an 11% reduction in coronary events (coronary heart disease death or myocardial infarction) (*P* = 0.16) and an 11% reduction in cardiovascular disease events (*P* = 0.04). After adjustment, the effects of fenofibrate on coronary events and cardiovascular disease events were 16% (*P* = 0.06) and 15% (*P* = 0.008), respectively.

**Conclusions/Significance:**

This novel application of a penalized Cox model for adjustment of a trial estimate of treatment efficacy incorporates evidence-based estimates for other therapies, preserves comparisons between the randomized groups, and is applicable to other long-term trials. In the FIELD study example, the effects of fenofibrate on the risks of coronary heart disease and cardiovascular disease events were underestimated by up to one-third in the original analysis.

**Trial Registration:**

Controlled-Trials.com ISRCTN64783481

## Introduction

A common problem in longer-term clinical trials comparing chronic treatments is that patients may start using additional active therapies during the course of the trial, which may confound the evaluation of the trial's target treatments [1], [2]. This is particularly the case when the uptake of such therapies differs between the treatment arms, potentially resulting in an underestimation of the direct benefit of the target treatment. Additional therapies may be given to patients as a result of new evidence emerging from other ongoing trials or because of changes in patient and clinician choice over time, and also to those responding poorly to initial treatments, as in some cancer trials [3]. Patients may discontinue trial treatments for similar reasons.

Conventional methods in clinical trials either use intention-to-treat analysis only or adjust for changes in treatment *after* randomization (such as in per-protocol analyses). The former may underestimate the true biological effect of treatment because of noncompliance, and the latter may be confounded by the differences between those patients who do and those who do not adhere to their randomized treatments or between those who do and those who do not take up other therapies. These latter analyses are prone to selection bias, in that they do not maintain the randomized structure of the comparisons [1], [4], [5].

This specific problem arose in the analysis of the 5-year Fenofibrate Intervention and Event Lowering in Diabetes (FIELD) trial — a large-scale trial of the lipid-modifying effects of fenofibrate compared with placebo in patients with type 2 diabetes mellitus [6]. The study design was pragmatic in evaluating the effect of fenofibrate on a background of usual medical care [6], [7]. This meant that in the light of new clinical circumstances or the emergence of new evidence, additional cardiovascular medicines, including statins and other lipid modifying treatments, could be commenced during the course of the trial. Methods have been proposed to account for noncompliance with randomized treatment [1], [8] (including instrumental variable analysis [9]–[11]), but these methods do not deal with the situation we encountered in FIELD of a large imbalance between the treatment groups in the proportion of patients who commenced *active nonstudy medications*. In the standard intention-to-treat analysis, unbalanced uptake of nonstudy treatment can attenuate the estimated effect of the study drug. Measuring the influence of the uptake of nonstudy medications requires estimates of the effect of these medications from sources external to the trial in question [1], as any estimates derived from within the trial are subject to selection bias. In this analysis, using FIELD as our example, we report a novel method for incorporating external evidence-based estimates to correct for this.

Adjustment for any dilution of the treatment effect caused by discontinuation of the randomized study drug by some patients was also examined by using a randomization-based efficacy estimator to adjust for nonadherence to study treatment [1].

## Methods

### FIELD Trial Design

FIELD was a randomized double-blind placebo-controlled trial in 9795 middle-aged to elderly people with type 2 diabetes mellitus [6], [7], [12] After a 16-week run-in period, patients were randomized to micronised fenofibrate (200 mg daily) or matching placebo and followed up through regular clinic visits in addition to usual care from their treating doctors for a planned median duration of 5 years.

During the course of the trial and before any unblinding of results, the trial's progress was monitored for rates of commencement of open-label lipid treatment, adherence to study treatment, and cardiovascular events (for both treatment groups combined). In the light of emerging evidence of the effectiveness of statin therapy, the increased uptake of statin treatment in the trial, and a lower than expected pooled event rate, the primary outcome, coronary heart disease (CHD) death, was revised in 2002 to CHD events (CHD death or myocardial infarction) [6]. The revised trial design was powered to detect a 22% reduction in CHD events (based on intention-to-treat analysis). This corresponded to a 27% reduction among those on treatment (based on a per-protocol analysis).

### Patient Population and Treatments

Patients with diabetes, with or without pre-existing cardiovascular disease or lipid abnormalities, were eligible, provided total blood cholesterol level at screening was 3.0 to 6.5 mmol/L, and either the triglyceride level was between 1.0 and 5.0 mmol/L or the total cholesterol/high-density lipoprotein cholesterol ratio was 4.0 or higher. Lipid values at screening were provided to the patients' doctors before randomization: all patients for whom any cholesterol-lowering treatment (including statins) was indicated at the start of the trial were ineligible. However, these (and other) medications could be commenced after randomization if the usual doctor considered it appropriate (for example, because of changed clinical circumstances) [12].

Cardiovascular medications were recorded at each follow-up visit (at least 6 monthly), as was adherence to study treatment.

### Cardiovascular Outcomes and Subgroups

The primary study endpoint was the first occurrence of CHD death or nonfatal myocardial infarction. Secondary outcomes included major cardiovascular events (CHD events, total stroke and other cardiovascular death combined), total cardiovascular events (major cardiovascular events plus coronary and carotid revascularization), CHD death, total cardiovascular deaths, stroke, and coronary and peripheral revascularization procedures.

The adjusted effect of fenofibrate on total cardiovascular events was examined within the main subgroups — men vs women, those aged <65 years vs those aged ≥65 years, and the presence vs absence of prior cardiovascular disease — to see whether differential uptake of other medicines by subgroup affected these comparisons.

### Estimates of Treatment Effect of Statins and Other Cardiovascular Drugs

The effectiveness of various medications in preventing cardiovascular events has been well established in several randomized controlled trials (Table S1) [13]–[20], with estimates of reductions in events in various settings ranging from 16% to 63%. The effect of combinations of drugs has, for the most part, been observed to be multiplicative on the basis of a similar relative risk reduction in randomized trials in the presence or absence of other drugs [14], [21].

In this analysis, evidence for the effectiveness of statins and other cardiovascular medicines was based on published systematic reviews of randomized trials of these therapies for diabetes populations and, in the absence of heterogeneity of treatment effects, for broader populations at risk of cardiovascular disease. Effects of the following medicines or classes of drugs were used in the adjusted analyses: statin drugs (simvastin, atorvastatin, pravastastin, any other statin); angiotensin-converting enzyme (ACE) inhibitors or angiotensin II receptor blockers; beta blockers; calcium-channel antagonists; diuretics; antiplatelet drugs (aspirin or other).

The estimate of the effect of statin use on subsequent cardiovascular events was based on the estimate of the Cholesterol Treatment Trialists' Collaboration's (CTTC) systematic overview of 14 large-scale randomized trials of statin therapy: a 21% reduction in cardiovascular events per mmol/L reduction in low-density lipoprotein (LDL) cholesterol [22]. Subgroup meta-analysis showed no heterogeneity of the statin treatment effect between those with and those without diabetes [23].

We estimated the absolute reduction in LDL cholesterol by statin therapy by applying the average percentage reduction in LDL cholesterol, estimated from a meta-analysis of 164 short-term randomized trials [24], to the LDL cholesterol levels of each treatment group (fenofibrate or placebo) in our cohort before they started any statin therapy. This average percentage reduction in LDL cholesterol was weighted according to the different statin drugs taken and the average dose of each used within each treatment group. The assumed event reduction (for each type of event) was then determined as the relative reduction in events per mmol/L reduction in LDL cholesterol multiplied by the average absolute reduction in LDL cholesterol.

For other cardiovascular drugs (ACE inhibitors, beta blockers, calcium-channel blockers, diuretics and antiplatelet drugs), a more simple, yet conservative, approach was taken. There was an assumed 20% reduction in the risk of any subsequent cardiovascular event due to the first nonstudy drug taken and a 15% reduction for each additional drug.

### Statistical Methods

All patients were included in the randomized comparisons, and analyses were by intention-to-treat. As specified in the published study protocol, the unadjusted primary analyses for cardiovascular events used standard log–rank methods without adjustment for covariates [25], and Cox proportional-hazards modelling was used to compute hazard ratios (HRs) and their 95% confidence intervals [26], [27].

Adjustment for the use of other cardiovascular medications used a penalized Cox model [27], for which the general formula for the hazard function at time *t* for patient *i* is:
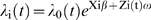
where *λ_0_(t)* is the baseline hazard function, 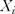 is the covariate indicator for treatment group ( = 1 for fenofibrate and 0 for placebo) and Z_i_(t) is the covariate vector indicating usage of cardiovascular disease medicines at time *t* for patient *i*. In this model *β* is the parameter for the treatment effect of fenofibrate (unconstrained coefficient), while **ω** is a vector of the assumed effects of other cardiovascular disease medicines (constrained coefficients). The HR from this model for the adjusted fenofibrate effect is estimated as exp (*β∧*).

When we adjusted for the effect of statins only, the constrained coefficient **ω** (offset) in this formula was set to the log of the HR for the effect of statin therapy within the treatment group. For example, the evidence-based effect of statins on total cardiovascular events was estimated to be 25% in those on placebo, and thus for a placebo patient, **ω** = log(0.75). In the case of adjustment for other cardiovascular medications, the value of the offset was calculated on the basis of the number and type of medicines taken by each patient at any time (20% reduction in risk from the first additional drug taken and a 15% reduction for each additional drug). The value of the offset in these cases was the addition of log(HR for statins), if the patient was treated with statins, plus log(0.8) for the first additional drug plus log(0.85) for each subsequent drug. This value changed as the patient's prescriptions changed. For example, a patient taking a statin, an ACE inhibitor and a diuretic would have an offset value of –0.673 (log (0.75×0.8×0.85)) only for the period he or she was on this combination of therapies.

The efficacy of fenofibrate in a fully adherent group was estimated, using randomization-based efficacy estimators or instrumental variable analysis, [1], [10] by adjusting for the nonuse of fenofibrate by the following approximation method:
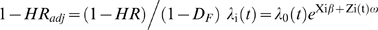
where *HR_adj_* is the adjusted HR estimate, *HR* the unadjusted estimated HR, and *D_F_* the proportion of patients discontinuing fenofibrate therapy averaged over the study period [28], [29]. An alternative version of this adjustment was undertaken in which *D_F_* was the average proportion discontinuing fenofibrate among patients having an event [1]. To avoid potential bias due to treatment decisions that might have been related to the event itself, we excluded from these calculations data from patients starting cardiovascular drugs within 1 month of the event.

All results were unadjusted for multiple comparisons. All analyses used SAS (version 9.1; SAS Institute, Inc. Cary, NC).

## Results

### Patient Characteristics and Use of Lipid-Modifying Therapies

Patient characteristics are shown in Table 1. Lipid-lowering therapy was commenced more often in the group assigned placebo than the group assigned fenofibrate (average use 17% vs 8%; *P*<0.001) and more often among the groups with prior cardiovascular disease, dyslipidemia or higher baseline LDL cholesterol levels (each *P*<0.001). The most common lipid-lowering therapy used was a statin (in 93% of patients), followed by a fibrate (6%), and other (2%).

**Table 1:** Use of the study drug and other medication (average % over 5 years) by treatment group and major subgroup in the FIELD study (*n* = 9795).

		Discontinuedstudy drug	Discontinued study drug	Started other lipid-lowering treatment*	Started other lipid-lowering treatment*
Subgroup	%	Placebo group	Fenofibrate group	Placebo group	Fenofibrate group
**Sex**					
Men	63	9	10	17	9
Women	37	10	11	18	7
**Age (years)**					
<65	60	9	9	17	8
≥65	40	10	12	18	9
**Previous CVD**					
Yes	22	11	14	23	14
No	78	9	9	16	7
**Hypertension**					
Yes	84	9	10	17	9
No	16	10	10	16	7
**Waist measurement**					
High†	68	10	10	17	9
Low	32	9	11	17	7
**Dyslipidemia**					
Yes‡	38	10	12	21	12
No	62	9	10	15	6
**HDL cholesterol (mmol/L)**					
High	41	9	9	15	6
Low§	59	10	11	19	10
**LDL cholesterol (mmol/L)**					
<3.0	45	9	11	11	6
3.0–3.5	29	9	10	17	9
>3.5 mmol/L	26	11	11	28	11
**All patients**	100	10	10	17	8

* Based on patients who took statins, resins, fibrates or other lipid-modifying drugs for at least 3 months.

† Men: ≥102 cm; women: ≥88 cm.

‡ Low HDL cholesterol plus high triglyceride (≥1.7 mmol/L).

§ <1.03 mmol/L for men, <1.29 mmol/L for women.

FIELD = Fenofibrate Intervention and Event Lowering in Diabetes; CVD = cardiovascular disease; HDL = high-density lipoprotein; LDL = low-density lipoprotein.

**Figure 1:**
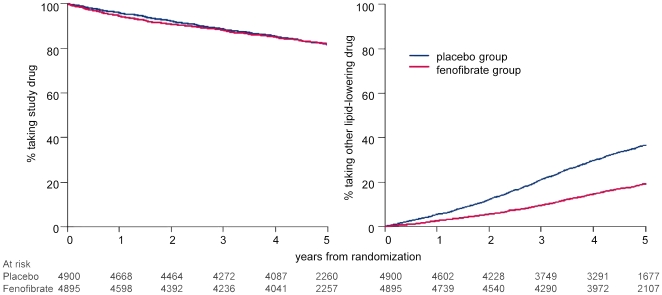
Time to discontinuing study medication or to starting other lipid-lowering treatment, by randomized group.

The discontinuation rate of study medication was similar in both randomized groups, steadily increased over time, and averaged 10% over the follow-up period of 5 years (Table 1 and Figure 1). The rate of discontinuation was somewhat higher than average among patients with prior cardiovascular disease or older age, but was similar for other major groupings. The discontinuation rate among those subsequently having a cardiovascular event was 8.7% in those assigned to placebo and 13.9% in those assigned to fenofibrate.

### Risk Factors for the Use of Lipid-Modifying Therapies

In a multivariable analysis of baseline risk factors in the placebo group, higher LDL cholesterol, lower high-density lipoprotein cholesterol, higher systolic blood pressure, lack of obesity, a previous CHD event, and country (Australia or New Zealand) each independently predicted a higher rate of commencing lipid-lowering therapy, particularly statins (Table 2). When classified according to a risk score for commencing lipid-lowering therapy, those patients with the highest scores had a significantly higher event rate than those with the lowest scores, indicating that patients at higher risk of cardiovascular disease events were much more likely to start statin therapy (Table S2).

**Table 2:** Risk factors for starting lipid-lowering therapy during the FIELD study.*

Risk factor	% of 4900 patients*	% using lipid-lowering therapy†	Adjusted HR (95% CI)‡	*P*
**Country**				<0.001
Finland	14	18	1.00	
Australia	62	36	2.03 (1.68–2.44)	
New Zealand	24	48	2.25 (1.85–2.75)	
**Clinical history**				
Prior myocardial infarction	5.2	51	1.50 (1.23–1.83)	<0.001
Prior angina	12	47	1.43 (1.24–1.66)	<0.001
Prior PTCA	1.3	57	1.58 (1.12–2.23)	0.01
BMI≥30 kg/m^2^	48	35	0.91 (0.83–1.00)	0.04
**Systolic blood pressure (mm Hg)**				0.003
≤130	27	35	1.00	
>130–140	25	36	1.14 (1.00–1.30)	
>140–150	24	38	1.22 (1.07–1.39)	
>150	24	37	1.27 (1.11–1.45)	
**LDL cholesterol (mmol/L)**				<0.001
<2.52	20	18	1.00	
2.52–<2.91	20	28	1.62 (1.34–1.96)	
2.91–<3.25	20	36	2.21 (1.84–2.65)	
3.25–<3.63	20	42	2.63 (2.20–3.14)	
≥3.63	20	57	4.23 (3.56–5.03)	
**HDL cholesterol (mmol/L)**				<0.001
<0.88	20	39	1.00	
0.88–<1.005	20	40	0.95 (0.83–1.10)	
1.005–<1.125	20	34	0.73 (0.63–0.84)	
1.125–<1.285	20	35	0.75 (0.65–0.87)	
≥1.285	20	34	0.78 (0.67–0.91)	

* Model derived by using the placebo group only.

† Patients who had started using statins, fibrates, resins or other lipid-lowering medications during the trial and had remained on them for at least 3 months in total.

‡ The initial variables were: sex, age, country, clinical history (myocardial infarction, stroke, angina, CABG, PTCA), smoking status, BMI, waist–hip ratio, systolic blood pressure, diastolic blood pressure, LDL cholesterol, HDL cholesterol, triglyceride.

FIELD = Fenofibrate Intervention and Event Lowering in Diabetes; HR = hazard ratio; CI = confidence interval; PTCA = percutaneous transluminal coronary angioplasty; BMI = body mass index; HDL = high-density lipoprotein; LDL = low-density lipoprotein.

### Use of Statin Therapy and Evidence of Reduction in Cardiovascular Disease Events

Among patients starting statin therapy, simvastatin and atorvastatin were most used (Table 3). On the basis of the meta-analysis by Law et al. [24] and the average daily dosage of statin, the percentage reduction in LDL cholesterol (average for both groups) was estimated as 44%, 33%, and 27% for those on atorvastatin, simvastatin and pravastatin, respectively. Among those who started statin therapy, the average LDL cholesterol before starting therapy was 3.31 mmol/L in the placebo group and 3.04 mmol/L in the fenofibrate group; the average absolute reductions in LDL cholesterol were estimated at 1.18 and 1.09 mmol/L, respectively.

**Table 3:** Average use of statins in FIELD and assumed effects on subsequent LDL cholesterol.

Drug	Treatment group	% started statin	Average dose (mg/day)	% reduction in LDL cholesterol*	Assumed change in LDL cholesterol (mmol/L)†
Atorvastatin	Placebo	6.1	20.5	43	−1.43
	Fenofibrate	2.8	21.2	44	−1.33
Simvastatin	Placebo	8.1	24.2	33	−1.09
	Fenofibrate	3.7	25.5	33	−1.01
Pravastatin	Placebo	2.4	28.9	26	−0.87
	Fenofibrate	1.4	30.0	27	−0.81
Other statin	Placebo	0.5	—	33	−1.09
	Fenofibrate	0.2	—	33	−1.00
Any statin	Placebo	16.1‡	—	—	−1.18 §
	Fenofibrate	7.9	—	—	−1.09 §

* Derived from meta-analysis of short-term randomized trials of statins [24].

† Calculated from the percentage reduction in LDL cholesterol applied to the average prior LDL cholesterol level in each treatment group for those patients who subsequently started lipid-lowering therapy.

‡ 93% of patients who started other lipid-lowering treatment took statins.

§ Based on a weighted average of LDL change for individual statins.

FIELD = Fenofibrate Intervention and Event Lowering in Diabetes; LDL = low-density lipoprotein.

The assumed effects of statin therapy on subsequent cardiovascular events were estimated as 27% and 25% reductions in CHD events and 25% and 23% reductions in cardiovascular events in the placebo and fenofibrate groups, respectively (Table 4). Very similar estimates were obtained when we applied the results from the CTTC overview of statin therapy for patients with diabetes [23] in a sensitivity analysis.

**Table 4:** Assumed effects of using statins (% relative risk reduction*) on subsequent cardiovascular (CVD) events in the FIELD study.

Type of CVD event	Placebo group†	Fenofibrate group†	All patients
CHD event	27	25	26
CHD death	22	21	22
Nonfatal MI	31	28	30
CVD death	20	18	19
Stroke	20	18	19
Revascularization	28	26	27
Any CVD event	25	23	24

* Estimates of event reduction per mmol/L change in LDL cholesterol were derived from the Cholesterol Treatment Trialists' overview of statin therapy [22].

† Assumed absolute change in LDL cholesterol from statin use: −1.18 mmol/L in the placebo group and −1.09 mmol/L in the fenofibrate group.

FIELD = Fenofibrate Intervention and Event Lowering in Diabetes; CHD = coronary heart disease; MI = myocardial infarction.

### Use of Other Cardiovascular Medicines and Evidence from Randomized Trials of Cardiovascular Event Reduction

Use of other cardiovascular drugs was well balanced between treatment arms at baseline (Table 5). These treatments increased over the course of the trial. By the close of the study, antiplatelet therapy was used by half the patients. Use of ACE inhibitors, beta-blockers and diuretics was slightly more common among patients assigned placebo than those assigned fenofibrate (each *P*<0.05).

**Table 5:** Percentages of patients using other cardiovascular drugs at baseline and study close, by randomized group, in the FIELD study.

	Baseline	Baseline	Study close	Study close
Type of drug	Placebo (n = 4900)	Fenofibrate (n = 4895)	Placebo (n = 4900)	Fenofibrate (n = 4895)
Any antiplatelet	29	29	51	50
Aspirin	29	29	47	46
Other antiplatelet	0.6	0.3	4	4
Angiotensin-converting enzyme inhibitor	34	33	48	45
Angiotensin II receptor antagonist	5	5	20	20
Beta-blocker	14	15	26	24
Calcium antagonist	19	20	27	26
Nitrate	6	5	12	11
Diuretic	15	15	24	21

FIELD = Fenofibrate Intervention and Event Lowering in Diabetes.

### Effect of Fenofibrate on Cardiovascular Disease Events

**Figure 2:**
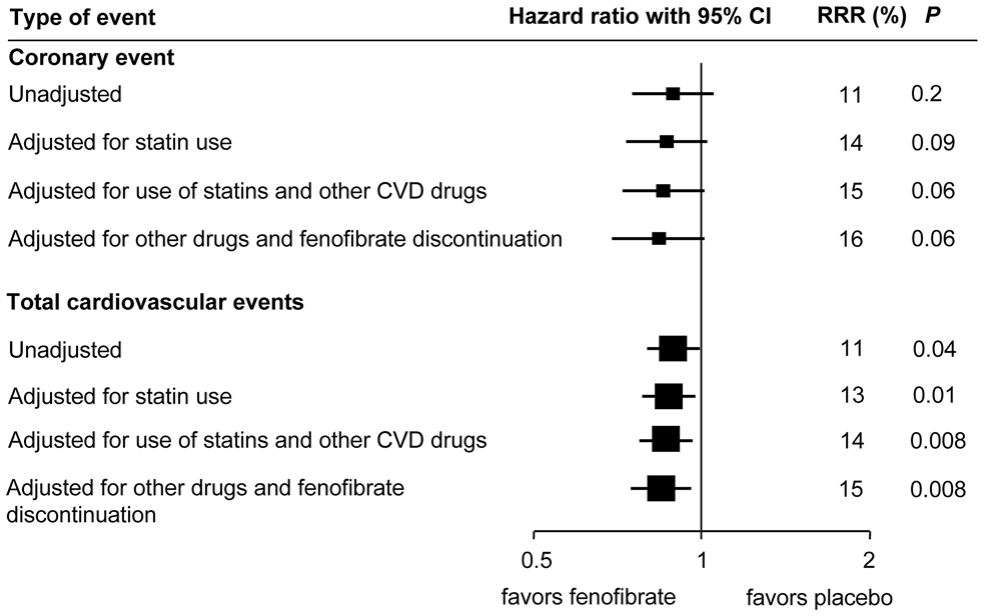
Effects of fenofibrate on events, with and without adjustment for use of statins and other drugs (RRR = relative risk reduction).

Before any adjustment for other drugs, fenofibrate was associated with a significant 11% relative risk reduction in total cardiovascular disease events (*P = *0.04) and a nonsignificant 11% reduction in the primary outcome, CHD events (*P = *0.2) (Table 6, Figure 2). After adjustment for the effect of statin therapy and other medicines on subsequent cardiovascular disease events, the effect of fenofibrate was moderately larger (Table 6). After the additional adjustment for discontinuation of fenofibrate therapy, efficacy estimates moderately improved to a 15% reduction in cardiovascular events (*P = *0.008) and a 16% reduction in CHD events (*P = *0.06). About two-thirds of the change in effect due to adjustment for other medicines can be explained by statin use alone (Figure 2). After adjustment for the use of other medicines and discontinuation of fenofibrate, the effect of fenofibrate on nonfatal myocardial infarction increased from 24% to 30%, on stroke from 10% to 14%, and on revascularization from 20% to 25%. The previously reported nonsignificant increase in cardiovascular disease deaths was reduced from 11% to 8%.

**Table 6:** Treatment effects of fenofibrate unadjusted and adjusted for the use of other CVD drugs in the FIELD study.

Outcome	No. events	Unadjusted RRR (95% CI)	*P*	RRR adjusted for use of statins and other CVD drugs (95% CI)	*P*	RRR additionally adjusted for fenofibrate discontinuation* (95% CI)	*P*
CHD event	544	11 (−5 to 25)	0.2	15 (−1 to 28)	0.06	16 (−1 to 31)	0.06
CHD death	203	−19 (−57 to 10)	0.2	−14 (−51 to 13)	0.3	−16 (−56 to 15)	0.3
Nonfatal MI	365	24 (6 to 38)	0.01	27 (10 to 41)	0.003	30 (11 to 45)	0.003
CVD death	267	−11 (−41 to 13)	0.4	−7 (−36 to 16)	0.6	−8 (−40 to 18)	0.6
Stroke	333	10 (−12 to 27)	0.4	12 (−9 to 29)	0.2	14 (−10 to 33)	0.2
Revascularization	851	20 (8 to 30)	0.002	22 (11 to 32)	<0.001	25 (13 to 36)	<0.001
Any CVD event	1295	11 (1 to 20)	0.04	14 (4 to 23)	0.008	15 (4 to 25)	0.008

* Adjusted for uptake of statins and other drugs and for discontinuation of fenofibrate.

FIELD = Fenofibrate Intervention and Event Lowering in Diabetes; CVD = cardiovascular disease; RRR = relative risk reduction (%); CI = confidence interval; CHD = coronary heart disease; MI = myocardial infarction.

In a sensitivity analysis using the approach suggested by White [1] (using the discontinuation rate only among those having an event), the fully adjusted estimates of the effect of fenofibrate were a 16% reduction in cardiovascular events and a 17% reduction in CHD events.

### Treatment Effects Within Subgroups

**Figure 3:**
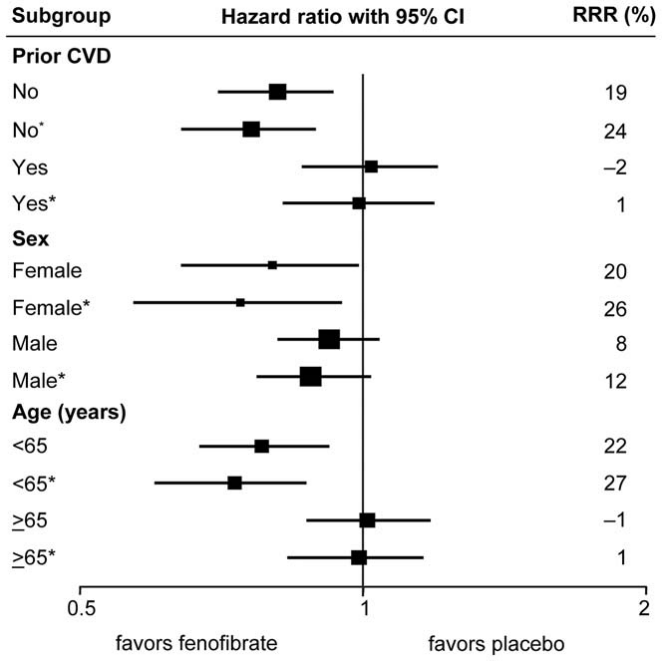
Effects of fenofibrate on cardiovascular events, by major subgroup. *Adjusted for use of other cardiovascular drugs and discontinuation of fenofibrate. RRR = relative risk reduction.

Treatment effects within major subgroups are shown in Figure 3 and Table S2. As previously reported, the treatment effect of fenofibrate was apparently larger among patients without prior cardiovascular disease than those with, and among patients aged under 65 years than those aged at least 65 years, as demonstrated by the interaction *P* values. Such tests for heterogeneity were nominally statistically significant, but only when not adjusted for the multiple subgroup comparisons. The apparent heterogeneity remained similar after adjustment for the differential use of statins and other cardiovascular medicines, but these differences became less significant when other baseline covariates were also adjusted for. Within each subgroup, the HRs became somewhat lower, reflecting the greater effect of fenofibrate after adjustment. There was no consistent pattern of a fenofibrate effect on cardiovascular events by quintile of risk of statin use, either before or after adjustment for use of other medicines (Table S2).

## Discussion

Our method for adjusting a trial result for other active treatments is novel. It extends established methods by adjusting for use of nonstudy medication in a fashion that is not subject to selection bias. As such, this can be thought of as an adjusted intention-to-treat analysis, which better determines the underlying true effect of the study treatment and also estimates an effect for a fully adherent patient group, while avoiding the biases inherent in a per-protocol analysis. In the example of the FIELD study, the likely true effects of treatment were underestimated by more than one-third in the original simple intention-to-treat analysis.

Conventional methods to adjust for both nonadherence and *nonstudy* treatments after randomization have been subject to selection bias [1], [4], [5] and higher false-positive rates. Apparent differences in outcomes may be driven by selection of patients for inclusion in analysis, rather than by treatment effects.

Methods that adjust the intention-to-treat analyses according to rates of noncompliance and other factors — randomized-based efficacy estimation methods — avoid selection bias by basing the analyses on the original groups [1], [3], [8]–[11], [28]–[32]. Approaches to date have largely been restricted to adjustment for the use or nonuse of the *trial* medicines. In particular, instrumental variable analysis [1], [9]–[11] can estimate average treatment efficacy among compliers, or among all patients under the assumption of full adherence to randomized treatment. Estimates are valid under such an assumption, but, unlike those from per-protocol analyses, do not account for drop-outs being sicker or healthier than those continuing on medication, and these methods do not address the uptake on *nonstudy* medicines. We adopted a simplified form of this approach for adjusting for the discontinuation of fenofibrate.

Our method of adjustment for use of nonstudy medicines —applying external randomized trial evidence for these drugs —uses a randomization-based efficacy estimate that is not subject to selection bias. The method applies the same relative risk reduction (as estimated in other randomized trials) to all patients after they commence such therapies. This assumes that the same relative risk reduction from these treatments would apply to a broad cross-section of patients. This appears to be the case in our example on the basis of evidence from several randomized trials showing no significant heterogeneity of treatment effect across a wide range of subgroups [14,21–23].

There are several examples of placebo-controlled trials in which the uptake of other active treatments differed by randomized group, and where this may have contributed to an underestimation of treatment effect or failure to detect a significant effect for some outcomes. These include trials of antihypertensive therapy or antiplatelet therapies to prevent vascular events. In trials such as Reduction of Endpoints in NIDDM with the Angiotensin II Antagonist Losartan (RENAAL) [33], the Study on Cognition and Prognosis in the Elderly (SCOPE) [34], the Jikei Heart Study [35], and Action in Diabetes and Vascular Disease (ADVANCE) [36], the use of other antihypertensive agents was greater in the placebo group, leading to a likely underestimation of the underlying effect of the trial treatment, and the possibility of missing effects on some outcomes. In the Clopidogrel in Unstable Angina to Prevent Recurrent Events (CURE) trial [37], which evaluated clopidogrel in acute coronary syndromes, use of thrombolytic agents and glycoprotein IIb/IIIa inhibitors was slightly greater in the placebo group. The effects of nontrial treatments on estimates may have been small in some cases, but their effects can be more directly assessed by our approach.

While we support the use of unadjusted intention-to-treat analyses as the primary analysis in randomized trials, it should be recognized that this may underestimate the efficacy of treatment as applied in practice. In our example, the primary results of the FIELD trial based on an intention-to-treat analysis of all patients showed smaller effects of fenofibrate on cardiovascular events than expected in the trial design. The trial was well powered to detect a true 27% reduction in cardiovascular events, corresponding to an approximate 22% reduction in the intention-to-treat analysis. The observed effects of fenofibrate were substantially smaller than this, probably in part because the average true effect of the drug is more modest, but also because the results of the intention-to-treat analyses were attenuated by about one-third, owing to substantial uptake during the trial of other medicines, particularly statins, together with the discontinuation of fenofibrate by some patients.

The adjusted analyses suggest that plausible treatment effects of fenofibrate in this setting are a 15% reduction in all cardiovascular disease events, a 16% reduction in major CHD events (the primary endpoint), and 30% reduction in nonfatal CHD events, all of which would make the value of such treatment more compelling. A 15% reduction would correspond to an absolute reduction in risk of about 2% or a number needed to treat of 50 patients over 5 years to prevent a major cardiovascular disease event. The adjusted analyses not only indicate a moderately larger treatment effect but also provide strong statistical evidence for a more substantial treatment effect than can be claimed using an intention-to-treat analysis. These analyses also allow some examination of whether the different uptakes of other cardiovascular disease medicines may have been a factor in the apparent variation in treatment effect within subgroups. Statin therapy was more likely to be used by patients with prior cardiovascular disease than not and by patients with more abnormal (than normal) lipid profiles. However, adjustment for the use of statins and other cardiovascular medicines did not appreciably alter the possible heterogeneity for these subgroups, so this does not explain the apparently different treatment effects. As discussed in more detail elsewhere [1], heterogeneity across some subgroups is still consistent with a chance finding and may relate, in part, to some differences in other baseline characteristics.

The methods have some limitations. These include: 1. the use of the same assumed treatment effect for each drug applied to each individual patient; 2. the use of evidence from different settings than might apply exactly to the trial setting of interest; and 3. the post-hoc nature of the assumptions made in this example, such as the source of external evidence. The post-hoc nature is not a limitation of the method itself, as these concerns could be addressed by building these approaches into the final analysis plan of future trials before unblinding. Also, one could introduce some randomness to the estimates used. In this analysis we chose conservative assumptions or undertook sensitivity analyses of alternative assumptions. For example, we assumed less than a fully multiplicative model when considering multiple drugs in the same patient, and variation in our assumptions resulted in similar conclusions.

Further refinements to the general approach are possible by: 1. using individual estimates of risk reduction based on a particular drug and dose (rather than applying the average risk reduction to all patients); and 2. considering a random variation in the size of the treatment effect for individual patients. It is recognized that the latter will lead to higher final *P* values.

Direct validation of this example in another randomized trial will not be possible, as a trial of the same type is no longer possible. However, it will be of interest to see, in the next 6 months, the results of the Action to Control Cardiovascular Risk in Diabetes (ACCORD) trial [38], which is evaluating the additional effect of fenofibrate on a background of all patients receiving statin therapy.

In conclusion, we have adjusted for the effects of an intervention by applying a new method that is not subject to selection bias, that provides an estimate of the underlying treatment effect and that takes into account both adherence to study treatment and the differential use of other nonstudy medicines. The approach suggests a moderate but real underestimate of the effects of fenofibrate on the prespecified cardiovascular outcomes of the FIELD study, which would make a stronger case for using such therapy. The adjusted results should provide more reliable estimates for future clinical decision making. The application of evidence-based estimates related to the use of other nonstudy (cardiovascular) medicines, as described in this setting, may be relevant to many long-term clinical trials, and the approaches adopted here should therefore have wide application and will be especially valuable where differential changes in usual care between treatment arms occur.

## Supporting Information

Table S1 Assumed relative reduction (%) in major cardiovascular events from other cardiovascular medication.
(0.03 MB DOC)

Table S2 Hazard ratios for the effects of fenofibrate on cardiovascular (CVD) events unadjusted and adjusted for the use of statins and other CVD drugs within major subgroups and according to risk of starting CVD drugs.
(0.06 MB DOC)
