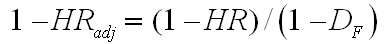# Correction: A Novel Method to Adjust Efficacy Estimates for Uptake of Other Active Treatments in Long-Term Clinical Trials

**DOI:** 10.1371/annotation/54433693-04e0-4f30-9a99-38fe3c5bb16b

**Published:** 2010-01-25

**Authors:** John Simes, Merryn Voysey, Rachel O'Connell, Paul Glasziou, James D. Best, Russell Scott, Christopher Pardy, Karen Byth, David R. Sullivan, Christian Ehnholm, Anthony Keech, for the FIELD Study Investigators

In the Methods section, in the fourth paragraph of the subsection "Statistical Methods," the equation was incorrect. Please view a correct version of the equation at :