# Supplementary material for: A Novel Method to Adjust Efficacy Estimates for Uptake of Other Active Treatments in Long-Term Clinical Trials
Source: PLoS One. 2010 Jan 8;5(1):e8580. doi: 10.1371/journal.pone.0008580 (PMC2798963; doi:10.1371/journal.pone.0008580)
Supplement: Table S1 — Assumed relative reduction (%) in major cardiovascular events from other cardiovascular medication. (0.03 MB DOC) [file pone.0008580.s001.doc]

| Cardiovascular drug | Estimated relative risk reduction for cardiovascular events (%) | **Reference** |
| --- | --- | --- |
| Antiplatelet or aspirin | 22 | Antithrombotic Trialists’ Collaboration [13] |
|  | 16 to 32 | Wald, Law [14] |
| Beta-blocker | 20 (hypertension)  27 (secondary prevention) | Collins et al.[15]  Yusuf et al.[16,17] |
| Angiotensin-converting enzyme inhibitor or angiotension II receptor antagonist or both | 21 | Blood Pressure Lowering Treatment Trialists’ Collaboration [18] |
| Calcium antagonist | 28 | Blood Pressure Lowering Treatment Trialists’ Collaboration [18] |
| Diuretic | ~20 | Collins et al. [15]  Law et al. [19] |
| Blood pressure lowering with diuretic or beta-blocker or both | 16 to 38 | Collins and MacMahon [20] |
|  | 46 to 63 | Law et al. [19] |
