# Supplementary material for: A Novel Method to Adjust Efficacy Estimates for Uptake of Other Active Treatments in Long-Term Clinical Trials
Source: PLoS One. 2010 Jan 8;5(1):e8580. doi: 10.1371/journal.pone.0008580 (PMC2798963; doi:10.1371/journal.pone.0008580)
Supplement: Table S2 — Hazard ratios for the effects of fenofibrate on cardiovascular (CVD) events unadjusted and adjusted for the use of statins and other CVD drugs within major subgroups and according to risk of starting CVD drugs. (0.06 MB DOC) [file pone.0008580.s002.doc]

|  |  | **CVD event rate (%)** | | **CVD event rate (%)** |  |  |  |  |
| --- | --- | --- | --- | --- | --- | --- | --- | --- |
| **Subgroup** | **% of patients** | **Placebo group** | | **Fenofibrate group** | **Unadjusted HR (95% CI)** | ***P*** | **Adjusted HR* (95% CI)** | ***P*** |
| Sex |  |  | |  |  |  |  |  |
| Women | 37 | 9.6 | | 7.7 | 0.80 (0.64–0.99) | 0.04 | 0.74 (0.57–0.95) | 0.02 |
| Men | 63 | 16.6 | | 15.4 | 0.92 (0.81–1.04) | 0.2 | 0.88 (0.77–1.02) | 0.09 |
| Interaction *P* |  |  | |  |  | 0.3 |  | 0.2 |
| Age |  |  |  | |  |  |  |  |
| <65 years | 60 | 11.6 | 9.2 | | 0.78 (0.67–0.92) | 0.003 | 0.73 (0.60–0.87) | <0.001 |
| ≥65 years | 40 | 17.4 | 17.4 | | 1.01 (0.87–1.18) | 0.9 | 0.99 (0.83–1.16) | 0.9 |
| Interaction *P* |  |  |  | |  | 0.02 |  | 0.02 |
| CVD* |  |  |  | |  |  |  |  |
| No | 78 | 10.8 | 8.9 | | 0.81 (0.70–0.93) | 0.004 | 0.76 (0.64–0.89) | <0.001 |
| Yes | 22 | 25.1 | 25.5 | | 1.02 (0.86–1.20) | 0.9 | 0.99 (0.82–1.19) | 0.9 |
| Interaction *P* |  |  |  | |  | 0.05 |  | 0.04 |
| **Risk for statin use (quintiles)** |  |  |  | |  |  |  |  |
| 1 Low | 20 | 9.1 | 8.7 | | 0.94 (0.70–1.26) | 0.7 | 0.91 (0.65–1.27) | 0.6 |
| 2 | 20 | 11.1 | 10.8 | | 0.96 (0.74–1.26) | 0.8 | 0.92 (0.68–1.25) | 0.6 |
| 3 | 20 | 13.4 | 10.0 | | 0.73 (0.56– 0.94) | 0.02 | 0.68 (0.50– 0.92) | 0.01 |
| 4 | 20 | 15.6 | 14.1 | | 0.91 (0.72–1.15) | 0.4 | 0.86 (0.66 –1.12) | 0.3 |
| 5 High | 20 | 20.2 | 19.3 | | 0.96 (0.79–1.17) | 0.7 | 0.91 (0.72–1.13) | 0.4 |
| Interaction *P* |  |  |  | |  | 0.5 |  | 0.6 |

* Analyses were adjusted for statin use, other drugs and discontinuation rates. Interaction *P* values were adjusted for statin use and other drugs only. If these interactions are also adjusted for other baseline covariates, then interaction *P*=0.07 for prior CVD subgroups and *P*=0.02 for age subgroups.

† Any self-reported history of myocardial infarction, angina, coronary artery bypass grafting, percutaneous transluminal coronary angioplasty, stroke, coronary revascularization or peripheral vascular disease

HR=hazard ratio; CI=confidence interval
